# Distributed Cooperative Regulation for Multiagent Systems and Its Applications to Power Systems: A Survey

**DOI:** 10.1155/2014/139028

**Published:** 2014-08-27

**Authors:** Jianqiang Hu, Yaping Li, Taiyou Yong, Jinde Cao, Jie Yu, Wenbo Mao

**Affiliations:** ^1^School of Automation, Southeast University, Nanjing 210096, China; ^2^China Electric Power Research Institute, Nanjing 210003, China; ^3^Research Center for Complex Systems and Network Sciences, Department of Mathematics, Southeast University, Nanjing 210096, China; ^4^Department of Mathematics, Faculty of Science, King Abdulaziz University, Jeddah 21589, Saudi Arabia; ^5^School of Electrical Engineering, Southeast University, Nanjing 210096, China

## Abstract

Cooperative regulation of multiagent systems has become an active research area in the past decade. This paper reviews some recent progress in distributed coordination control for leader-following multiagent systems and its applications in power system and mainly focuses on the cooperative tracking control in terms of consensus tracking control and containment tracking control. Next, methods on how to rank the network nodes are summarized for undirected/directed network, based on which one can determine which follower should be connected to leaders such that partial followers can perceive leaders' information. Furthermore, we present a survey of the most relevant scientific studies investigating the regulation and optimization problems in power systems based on distributed strategies. Finally, some potential applications in the frequency tracking regulation of smart grids are discussed at the end of the paper.

## 1. Introduction

The regulating of collective behaviors for multiple interacting units is of great importance for networked interconnected systems in practical engineering [1]. Because of the development of communication technologies, the past decade has witnessed a remarkable increase in the investigation of cooperative control of events in a multiagent system, where local interactions among the events can emerge some captivating phenomena, such as synchronization [2], consensus [3, 4], swarming [5], flocking [6], and rendezvous [7]. This does not come as a surprise since such cooperative phenomenon lies in the heart of many real scenarios, ranging from some natural phenomena (school of fish, bird flock, and herding) to physics/biology and social networks, as well as engineering applications designed by networked control systems or coordination control problems among multiple subsystems.

**Figure 1:**
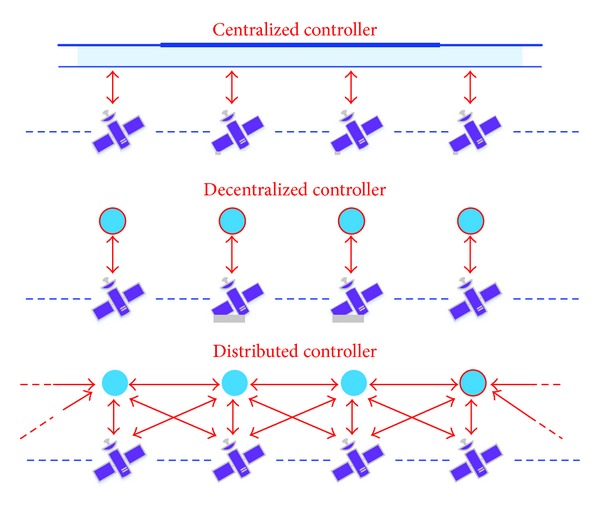
Three kinds of control strategies [8].

Generally, for a large-scale multiagent system, there are always three kinds of control strategies: centralized control, decentralized control, and distributed control. The main ideas about these control strategies can be seen from Figure 1. Centralized and decentralized controls are always employed in the practical engineering applications, such as in the automatic generation control (AGC) of the power system; the participation rate for each AGC unit is received at the terminal station from the dispatching center (in a centralized way) and grid friendly household appliances which can reduce the consumption of active power by local frequency detection of the power system (in a decentralized way). While for some application fields distributed control is better than the centralized control and decentralized control in terms of easy implementation, low complexity, high robustness, and good scalability, such as in the formation flying of satellites, it is costly and inefficient to control every satellite by a way of centralized control. The same is valid for underwater repair done by multiple underwater robots.

Cooperative control studies can be traced back to the synchronization control of the master-slave system [9], where there is a communication link between the node of the master system and the node of the slave system, which is also known as the drive-response system. Along with the development of communication technologies and the increasing number of the controlled plants, distributed cooperative control has become popular in recent years. As one kind of basic coordination problem, cooperative tracking is an interesting research topic subjected to one leader or multiple leaders. In fact, most of the practical problems are affiliated to tracking control problems subjected to one or multiple leaders, such as in power systems, the tracking of the expected regulation of the active and reactive power outputs of multiple photovoltaic (PV) generators [10], and the tracking of the reference voltage/frequency for the interconnected distributed generation (DGs) [11].

When there is only one leader and multiple followers in the multiagent network, the corresponding tracking problem is affiliated to consensus tracking or pinning consensus; when there are multiple leaders and multiple followers in the multiagent network, such a tracking problem is the containment control. For a multiagent system, consensus tracking control has been introduced in Hong et al. [12], where the state of the leader is time varying and not measured; in order to track such a leader, a neighbor-based local controller together with a neighbor-based state estimation rule was proposed for each autonomous agent such that all agents could follow the leader. Dimarogonas et al. [13] introduced a leader-based containment control strategy for multiple unicycle agents, where the leaders could converge to a desired formation and the followers could converge to the convex hull of the leaders' final positions.

This paper reviews some recent progress in leader-following tracking control, mainly the pinning consensus problem with a single leader and containment control problem with multiple leaders and its applications in power systems. The rest of this paper is organized as follows. We start by introducing the pinning consensus (leader-following consensus) problem in Section 2.  Section 3 provides the basic description about the multiple objective tracking problem for leader-following systems (containment control). Furthermore, the methods on how to choose the pinned nodes are summarized in Section 4.  Section 5 discusses some related studies on the regulation and optimization dispatch problems in the area of power systems by distributed strategies. Some potential applications for tracking control in power systems are discussed in Section 6.

The basic preparation for cooperative control is the communication network, so it is reasonable to introduce some fundamental concepts about networks as follows.

A directed graph (or digraph) *G* = (*V*, *E*) is used to represent the communication topology in a networked multiagent system, where *V* = {1,2,…, *N*} is the finite set of the agents and *E* is the set of edges. The edge *e*_*ij*_ = (*i*, *j*) ∈ *E* indicates that the agent *j* can receive the information from the agent *i*. A graph with the property that *e*_*ij*_ ∈ *E* implies *e*_*ji*_ ∈ *E* is said to be undirected. *G* is called strongly connected if between any pair of distinct nodes *i*, *j* ∈ *V* there is a directed path from node *i* to node *j*, and *G* is said to be weakly connected if replacing all of its directed edges with undirected edges appears to be a connected undirected graph. A directed tree is a digraph, where every node, except the root, has exactly one parent node. A spanning tree of *G* is a directed tree whose node set is *V* and whose edge set is a subset of *E*. For a digraph *G*, the adjacency matrix *A* ∈ *R* ^*N*×*N*^ is defined as *a*_*ij*_ ≥ 0, in which *a*_*ij*_ = 1⇔*e*_*ji*_ = (*j*, *i*) ∈ *E*, while *a*_*ij*_ = 0 if *e*_*ji*_ ∉ *E*, and it is further required that self-links are not allowed; that is, *a*_*ii*_ = 0. The Laplacian matrix *L* is defined as *L* = *D* − *A*, where *D* is a diagonal matrix with *d*_*ii*_ = ∑_*j*≠*i*_ *a*_*ij*_.

## 2. Pinning Consensus of Multiagent Systems

**Figure 2:**
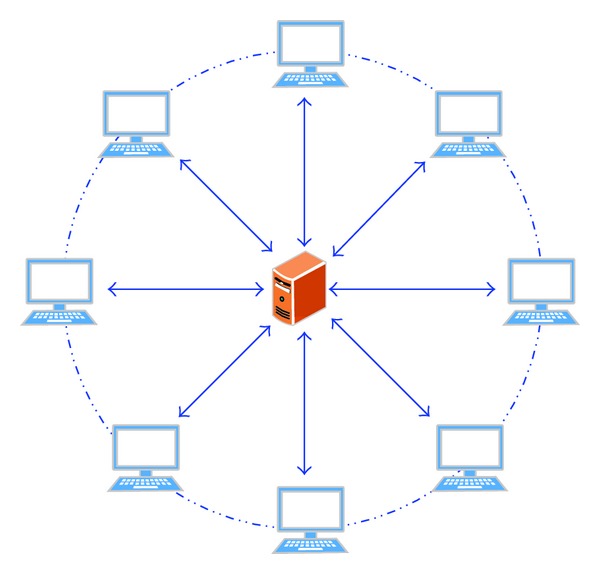
The communication structure for centralized control.

For a multiagent tracking network, the communication structure denotes the direction of information flow, by which agents are connected to each other. Pinning consensus means there are one leader agent and multiple followers in the system and to design a control strategy such that the followers can track the leader. The objective information can be viewed as the root node for the communication network; if there exists a directed path from the root to each agent, then all the agents can track the objective successfully, that is, the simplest star-network topology (see Figure 2); all units are pinned by the control center for the objective information.

Recently, distributed pinning control becomes very popular due to their flexibility and computational efficiency based on the sparse communication network. In fact, such an idea comes from the pinning synchronization of complex dynamical networks. Pinning control can be found in the literature [14, 15], where the authors turned to seek the minimum density of controllers for controlling the spatially extended chaotic systems. With the discoveries of the small-world network [16] and the scale-free network [17], complex networks have witnessed unprecedented developments in various fields, and the discussions of synchronization problems for complex networks have been extensively launched. However, in practice, the state variables of some network nodes are not observable or measured. Therefore, it is necessary to investigate the possibility of pinning a coupled network by adding controllers to those nodes, which can be measured or controlled.

### 2.1. Consensus of Multiagent Systems

One of the basic tasks in a multiagent system is the consensus of each agent's state. Consensus control has been subject to fundamental research as well as being an important theoretical preparation for other cooperation control problems, such as robotic coordination, distributed computation, and satellite formation flying. Generally, distributed consensus protocols are designed for the multiagent system. In such a distributed mode, each agent perceives the information of its neighboring agents and then responds according to the consensus protocol in real time, under which consensus can be achieved.

In a system of *N* agents, each characterized by a state variable *x*_*i*_(*t*) ∈ *R* ^*n*^ subject to a control input *u*_*i*_(*t*) ∈ *R* ^*n*^, is given as follows:

(1) $$\begin{array}{c} {\dot{x}}_{i}\left(t\right)=Ax_{i}\left(t\right)+Df\left(t,x_{i}\left(t\right)\right)+Bu_{i}\left(t\right), \end{array}$$

where *i* = 1,2,…, *N*.

Consensus control means to design a distributed communication protocol such that the state of each agent can reach agreement as *t* → *∞*; that is,

(2) $$\begin{array}{c} \underset{t\to \infty }{\lim}||x_{i}\left(t\right)-x_{j}\left(t\right)||=0,\;\forall i,j=1,2,\ldots ,N. \end{array}$$

Given a communication topology *G* for the *N* multiagent system with the adjacency matrix *A* = (*a*_*ij*_)_*N*×*N*_ describing the interaction among agents, the consensus objective can be achieved by the following distributed consensus protocol:

(3) $$\begin{array}{c} u_{i}\left(t\right)=-c\overset{N}{\underset{j=1}{\sum }}a_{ij}K\left(x_{i}\left(t\right)-x_{j}\left(t\right)\right), \end{array}$$

where *i* = 1,2,…, *N*; and *c*, *K* are the coupling strength and the feedback gain matrix to be determined.

Under the consensus protocol, the networked multiagent system turns out to be the following vector form:

(4) x˙(t)=(IN⊗A)x(t)+(IN⊗D)f(t,x) −c(L⊗BK)x(t),

where *x*(*t*) = (*x*_1_^*T*^(*t*),…, *x*_*N*_^*T*^(*t*))^*T*^, *f*(*t*, *x*) = (*f*(*t*, *x*_1_)^*T*^,…, *f*(*t*, *x*_*N*_)^*T*^)^*T*^, and *L* is the Laplacian matrix of the communication topology *G*.

The rest is to analyze the consensus convergence and the convergence rate of the close-loop system (4) under fixed/switching undirected or directed communication topology and so on.

**Remark 1 .**

In fact, system (1) is quite general, since it covers the multiagent systems with integrator-type dynamics, linear dynamics, and nonlinear dynamics; meanwhile, first-order, second-order, and higher-order multiagent system are all possible [18, 19].

### 2.2. Pinning Originated from Complex Dynamical Networks

A closely related research topic to consensus is the synchronization of complex dynamical networks. The complex dynamical network is coupled with lots of dynamic nodes, and all the nodes will achieve synchronization spontaneously if the coupling strength is sufficiently large. However, in most practical cases, the coupling strength is small and the network needs to be controlled to a desired homogeneous trajectory. The* pinning control strategy* was proposed in [20] for the scale-free dynamical network, where specifically and randomly pinning schemes were employed to synchronize the dynamical network. Furthermore, Li et al. [21] proposed the concept of virtual control and studied the pining control problem for a complex dynamical network. The concept of* pinning controllability* had been introduced in [1] for general complex dynamical networks, where networks are defined in which two different layers of dynamical nodes coexist: the uncontrolled sites and the reference (controlled) ones. It has been shown that the latter plays the role of leading the whole network towards a given (desired) reference evolution. Chen et al. [22] proved that a single controller can pin a coupled complex network to a homogenous trajectory if the network coupling strength is sufficiently large. In [23], the authors established some sufficient conditions for global pinning controllability of a generic network of coupled oscillators to some desired solutions. The authors in [24] considered the stochastic pinning synchronization of coupled dynamical system with Markovian switching couplings, where the coupling matrix and pinning feedback gain follow a common Markovian switching sequence. So far, pinning synchronization for complex dynamical networks has been extensively addressed; for more details see [25–30] and references therein.

However, the challenging problem for pinning control is which nodes should be pinned and at least how many nodes should be pinned for undirected or directed network structures. Recently, Liu et al. [31] developed analytical tools to study the controllability of an arbitrary complex directed network based on the theory of maximum matching, which can identify the set of driver nodes with time-dependent control. The authors [32] further investigated the observability of complex systems. Yu et al. [33] investigated how to choose an optimal node to realize the pinning controllability of the complex network based on the cut graph theory. And recently, in [34], the optimal choice for the *k* (*k* < *N*) follower nodes to be pinned can be approximately solved by minimizing the maximal distance in the communication graph from the leader to the followers. Such a *k*-pinning problem has been transformed to a nonconvex optimization problem. The authors in [35] introduced an analytical approach to select leader agents, in order to minimize the total mean-square error of the follower agents in the presence of noisy communication links.

### 2.3. Consensus Tracking of Multiagent System

In a one-dimensional integrator multiagent system, by integrating the distributed consensus protocol to the node system, then the following coupled system appears:

(5) $$\begin{array}{c} {\dot{x}}_{i}=-\overset{N}{\underset{j=1}{\sum }}a_{ij}\left(x_{i}-x_{j}\right),\;i=1,2,\ldots ,N. \end{array}$$

In vector notation, the consensus protocol (5) takes the form $\dot{x}=-Lx$, where *L* is the corresponding Laplacian matrix of the communication topology *G*. Under such protocol, the multiagent system will achieve consensus on a common state; that is, all *x*_*i*_(*t*) will converge to a common value *x*_*∞*_ as *t* → *∞*. If the graph *G* has a directed spanning tree, then all the eigenvalues of Laplacian matrix *L* have nonnegative real parts and zero is an eigenvalue with the right eigenvector 1_*N*_ and the left eigenvector *ω* = {*ω*_1_,…, *ω*_*N*_}, where ∑_*i*=1_^*N*^ *ω*_*i*_ = 1, *ω*_*i*_ ≥ 0. It follows that the consensus subspace 1_*N*_ is exponentially stable, the consensus value is the weighted average of the initial sates *x*_*∞*_ = ∑_*i*=1_^*N*^ *ω*_*i*_ *x*_*i*_(0), and the rate of convergence is not worse than *Re*{*λ*_2_(*L*)}; that is, ||*x*_*i*_(*t*) − *x*_*∞*_||≤||*x*_*i*_(0) − *x*_*∞*_||*e* ^−*Re*{*λ*_2_(*L*)}*t*^.

However, the consensus value *x*_*∞*_ in the above formula is not always the expected final state in practice. In order to control the multiagent system converging to a given objective value, the distributed pinning consensus protocol is introduced. The so-called “distributed pinning control” means only a small fraction of nodes in the network are pinned by the control center to the objective trajectory and the rest of the nodes communicated with each other to achieve the expected networked tracking.

The following distributed pinning protocol is given in [36]:

(6) $$\begin{array}{c} {\dot{x}}_{i}=-\overset{N}{\underset{j=1}{\sum }}a_{ij}\left(x_{i}-x_{j}\right)-d_{i}\left(x_{i}-\theta \right), \end{array}$$

where *i* = 1,2,…, *N* and the pinning control gain *d*_*i*_ ≥ 0 and *d*_*i*_ = 0 indicates there is no control over the agent *i* and *θ* is an expected consensus state.

Furthermore, Song et al. [37] investigated pinning consensus problems for second-order nonlinear multiagent systems with general network topologies and addressed what kind of agents and how many agents should be pinned. Based on the method of model predictive control (MPC) and pinning control, the authors in [38] showed that the consensus performances (i.e., the convergence speed towards consensus) could be improved, as the pinning nodes can be used to provide an accurate future state trajectory prediction due to the availability of the objective information shared with these nodes. The authors in [39] investigated finite-time distributed consensus problem for multiagent systems using a binary consensus protocol and the pinning control scheme. By using the Lie algebra theory, a linear node-and-node pinning method was proposed in [40] to achieve a consensus for the case of a directed multiagent network which does not contain a directed spanning tree.

**Figure 3:**
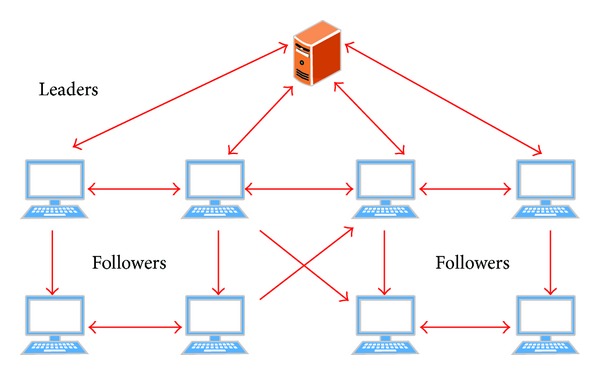
Pinning control structure of multiagent system.

In fact, leader-following consensus can be regarded as pinning consensus as well. Leader-follower models were introduced in the literature [12, 41, 42] where agents in the system can be categorized as leaders and followers. A leader can be viewed as an objective node which can perceive more information in order to guide the whole group, while a follower usually responds to the commands received from the leaders and other connected agents. If there exists only one leader in the network, the leader-following consensus is the general pinning consensus problem; see Figure 3.

Leader-follower models have been extensively studied in the literature in terms of controllability, formation, and target tracking [43–45]. The authors in [46] investigated the distributed tracking control for leader-follower multiagent system with measurement noises and directed interconnection topology. By using the iterative learning approach, tracking control problems were considered in [47] for multiagent systems which were described by both discrete-time and continuous-time models, where all agents in a directed graph were enabled to track a time-varying reference trajectory perfectly over a finite interval. Based on distributed discontinuous controllers with static/adaptive coupling gains, consensus tracking problem for linear multiagent systems was investigated such that all the followers could converge to the leader whose control input was nonzero and not available to any follower [48]. Xu et al. [49] considered the leader-following consensus of nonlinear multiagent system under switching topologies where the union of all the topologies was jointly connected. While for consensus tracking, the corresponding literature can be seen from [19, 50–52] and references therein. The pinning control strategy for multiagent systems can not only help us to better understand the mechanisms of natural collective phenomena, but also benefit applications in mobile sensor/robot networks [53–55].

## 3. Containment Control of Leader-Following Networks

**Figure 4:**
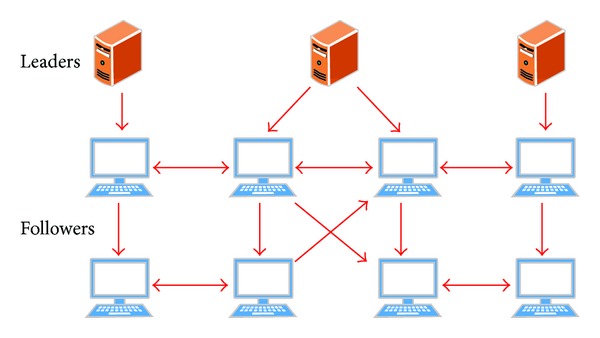
Containment control structure of leader-following multiagent system.

In the previous section, we mainly reviewed the leader-following consensus problems, where there is only one leader in the multiagent network. Containment control problem arises in the presence of multiple leaders and multiple followers in a multiagent network (see Figure 4) where its control objective is to drive all the followers into the convex hull spanned by the leaders. Motivated by numerous natural phenomena and some requirements of engineering applications, such as moving of migratory birds, earth monitoring satellites, and smart autonomous robots which can steer clear of obstacles, containment control has attracted substantial attention from various research communities.

Containment control is a combination of formation and rendezvous problem and as such it is of interest for UAV formation control, robot swarms, and attitude control of rigid bodies [13]. The authors in [56, 57] investigated the problem of driving a collection of mobile robots to a given target location in the context of partial difference equations by a decentralized control strategy for the followers, and the following agents would stay in the convex polytope spanned by dedicated leader agents, whose dynamics were given by a hybrid Stop-Go policy according to a decentralized formation control strategy. At this point, the leaders stop and let the followers settle back into the leader polytope before they start moving again. Inspired by [57], the authors in [58] furthermore considered a hierarchical model predictive control (MPC) structure for containment and distributed sensing in a leader-following multiagent architecture.

Containment control problems are further carried out in the single-/double-integrator agents with multiple stationary or dynamic leaders under fixed undirected/directed communication topologies [59–62] by distributed protocol. The authors [63] studied the containment control for continuous-time/discrete-time multiagent systems by proposing a new protocol which exploited the control input information of neighbors. While the containment control for linear multiagent system [64–66] has been considered recently, such as necessary and sufficient conditions were given in [67, 68] for discrete-time linear multiagent systems under both the state feedback and output feedback protocol cases. Robust *H*_*∞*_ containment controls for uncertain linear/nonlinear multiagent systems were further investigated recently in [69, 70]. On the other hand, the containment control for nonlinear multiagent systems has been investigated as well, such as containment for second-order nonlinear multiagent systems [71], finite-time containment for multiple Lagrangian systems [72], and multiple rigid body systems [73]. Distributed containment control problems were discussed in [74, 75] for nonlinear Euler-Lagrange systems with parametric uncertainties by introducing estimator or adaptive control law under a directed communication topology. Some other nonlinear multiagent system cases were studied in [76, 77].

On the other hand, switching communication topology is an attractive research area, which has been considered in the designing of the distributed protocol. As a kind of time-varying communication topology, switching always follows two different cases: random switching and Markov switching. Under the assumption that the union of undirected graphs is jointly connected, [78] considered the containment control problem in leader-following networks. By considering the dynamically/randomly switching topologies, [79] investigated the mean-square containment control for multiagent systems with transmission noises. The almost surely asymptotic containment was considered in [80] for a second-order multiagent system under the switching of a continuous-time irreducible Markov chain.

Generally, the containment control problem of a group of *N* nonlinear agents with the dynamic of *i*th agent can be described as follows:

(7) $$\begin{array}{c} {\dot{x}}_{i}=Ax_{i}+Df\left(t,x_{i}\right)+Bu_{i},\;i\in V_{F},\\ {\dot{x}}_{i}=Ax_{i}+Df\left(t,x_{i}\right),\;i\in V_{L}, \end{array}$$

where *x*_*i*_ ∈ *R* ^*n*^ and *u*_*i*_ ∈ *R* ^*m*^ are the state and the control input of *i*th agent and *V*_*F*_≜{1,…, *M*} and *V*_*L*_≜{*M* + 1,…, *N*} denote the followers' set and the leaders' set, respectively.

The distributed containment control protocol based on the real-time state information feedback for the followers in system (7) is given as

(8) $$\begin{array}{c} u_{i}=\alpha K\overset{N}{\underset{j=1}{\sum }}a_{ij}\left(x_{j}-x_{i}\right),\;i\in V_{F}, \end{array}$$

where *α* and *K* are the positive coupling strength and the feedback gain matrix for the protocol and *a*_*ij*_ is the element of the adjacency matrix of the directed communication topology *G*.

Protocol (8) is said to solve the containment control problem, if for any initial sates, all followers asymptotically converge to the convex hull spanned by the leaders. That is,

(9) $$\begin{array}{c} \underset{t\to \infty }{\lim}d\left(x_{i},\Omega \right)=0,\;\forall i\in V_{F}, \end{array}$$

where *Ω* = {∑_*i*=1_^*N*−*M*^ *α*_*i*_ *x*_*M*+*i*_∣*α*_*i*_ ≥ 0, ∑_*i*=1_^*N*−*M*^ *α*_*i*_ = 1} is the convex combination of the leaders' states.

In the above discussion (since 2010), there is a basic assumption that there is no communication among leaders. That is, the leaders are controlled in a centralized way. In fact leaders may communicate with each other so as to maintain a fixed formation or expected running space (as stated in [56]). Such a leader-following model presents a hierarchical structure with two layers, where the upper layer denotes the set of leaders and the lower layer represents the set of followers. The leaders communicate with each other such that a formation control objective can be achieved, and the followers communicate with each other such that they can converge to the formation based on the designed protocol. In [81], finite-time formation control has been considered for a group of agents where the global formation information was only available for navigational leaders; meanwhile, the other following agents regulated their positions by the local information in a distributed manner. Cooperative control has been investigated in [82] such that the leaders converged to a formation and the followers moved into the convex hull spanned by the leaders final positions under switching topologies. It deserves further investigations of distributed-distributed cooperative control in leader-following structures.

## 4. How to Choose the Pinning Nodes

As for a multiagent system, if the communication networks are prescribed, then the pinning consensus problem reduced to find the pinning nodes according to the network structure. On the other hand, pinning is related to the designing of an efficient communication network for the multiagent system according to the controllability and observability of each plant. When pinning a multiagent system, the most challenging problems are what kinds of nodes should be pinned and what is the minimum number of the pinned nodes. Up to now, some effective pinning schemes have been proposed for the directed/undirected complex networks, as reported in the literature.

When the communication network is undirected, one can choose randomly a set of nodes or specifically the most highly connected nodes to be pinned [20]. On the other hand, one can also utilize the following centrality methods to choose the pinning nodes, such as: local centrality [83], betweenness centrality [84, 85], closeness centrality [86], eigenvector centrality [87], subgraph centrality [88], PageRank centrality [89], LeaderRank centrality [90], and some applications [91, 92]. These centrality methods can be utilized to rank the nodes in the network according to the communication structure. High centrality nodes are always viewed as more important nodes in networks and they should be pinned first. However, the performance is evaluated and determined by the convergence rate under the minimal number of pinned nodes under the same pinning gains.

When the communication network is directed, how to select the appropriate pinned nodes is quite a challenging problem. In the literature, there have been several pinning strategies proposed. In [93], it is suggested to pin the roots of trees in a spanning forest of the interaction graph. Some high ControlRank (CR) nodes [94] have been chosen to be pinned. One can also make use of the closeness centrality of the weighted directed networks method [95] to decide whether the nodes should be pinned or not. In [96], the authors have proposed a selective method based on the pinned candidates set composed of the nodes whose out-degree is bigger than their in-degree. Illuminated by the idea in [96], it is considered that if there is a directed edge from the *i*th node to the *j*th node, the dynamical behavior of the node *j* will then be impacted by the dynamics of the node *i* to a great extent. The manipulating procedure is summarized as follows.

- For a given digraph with *G* ^(0)^ as its Laplacian matrix, the in-degrees and out-degrees of the nodes are defined as deg_in_(*i*) = ∑_*j*=1,*j*≠*i*_^*N*^ *G*_*ij*_^(0)^ and deg_out_(*i*) = ∑_*i*=1,*i*≠*j*_^*N*^ *G*_*ij*_^(0)^.
- Let deg_diff_(*i*) = deg_out_(*i*) − deg_in_(*i*)  (*i* = 1,2,…, *N*) be the degree differences of the digraph *G* ^(0)^.
- For the nodes with zero in-degrees, they should be pinned first since those nodes can be viewed as leaders of the network.
- Rearrange the remaining nodes in descending order according to their degree differences, and choose the pinning nodes according to the degree differences.

In summary, there exist many kinds of node ranking methods with respect to undirected networks and directed networks. However, the basic problem comes for a given large-scale communication network, which nodes should be pinned and at least how many nodes should be pinned, instead of checking each method one by one. This problem is a network optimization problem that remains to be further explored. Some recent progress with respect to optimization methods on how to choose the optimal pinning nodes can be seen in [33–35].

## 5. Cooperative Control in Power Systems

With the development of smart grids and the interconnection of multiple large-scale regional power grids, power system has increasingly developed into a supersized artificial network. Such a network consists of cross-coupled primary/secondary power equipments and is supported by advanced control technologies and efficient communications networks, which has formed a smart self-healing system. Traditionally, power system stability and control were accompanied by control strategies of centralized or decentralized control and rarely involved the distributed coordination control. Owing to the expansion of network scale and the increasing number of the controlled objects, distributed control gradually brings out its advantages in terms of better robust performance and lower cost of control. Recently, distributed control and distributed optimization are utilized to solve the emerged control and optimization problems in power systems.

### 5.1. Distributed Regulation

As for distributed cooperative control, consensus protocol and pinning tracking have been introduced in some application fields of power systems, especially in microgrids. Kim et al. [97] employed a cooperative control strategy to the islanded operation microsources and the energy storage system (ESS) and showed that such a control strategy could improve the control capability in the regulations of frequency and voltage. By selecting the incremental cost of each generation unit as the consensus variable, the authors in [98] introduced an incremental cost consensus (ICC) algorithm which was able to solve the conventional (centralized) economic dispatch problem of power systems in a distributed manner. Based on the cooperative control strategies, the regulation output was considered for multiple photovoltaic (PV) generators [99, 100] such that all the PVs had the same reserve ratio with respect to their maximum available power, and the regulation output for multiple distributed generators (DGs) was investigated in [101] in a distributed fashion such that these generators could be developed into a virtual power plant (VPP) in a distribution network. By input-output feedback linearization, the secondary voltage control problem was converted to a linear second-order tracking consensus problem [102] and it was shown that the distributed structure could improve the system reliability. Similarly, distributed control was introduced in distribution networks for coordinating multiple energy storage (ESUs) such that the ESUs's reactive power could be used for voltage support and the active power could be utilized in managing network loading [103]. Distributed frequency synchronization of multiple isolated microgrids was investigated in [104] under the smart grid communication infrastructure.

While most of previous publications focus on the distributed regulation problems for generating/storage units, the coordination problem among multiple controllable flexible loads in distribution networks has received much attention recently. By proposing a multiagent reinforcement learning algorithm and considering the nonlinear characteristics of power systems, Daneshfar and Bevrani [105] showed that load-frequency control (LFC) performance was improved compared with traditional proportional-integral (PI) controllers. A distributed multi-agent-based load shedding algorithm was proposed in [106], which could make efficient load shedding decision based on discovered global information, where the information discovery algorithm was given in a distributed way. Zhao et al. [107] proposed a decentralized optimal load control scheme via frequency measurement, where each load estimated the total mismatch between load and generation. Meanwhile, the inconsistencies of the estimations were mitigated by an average consensus algorithm. Furthermore, frequency regulation problem was considered in [108] by formulating an optimal load control problem. By proposing a market-based control and a multiagent distributed communication model, the optimal operation problem was considered in [109] for price-response controllable loads in electrical distribution network. For an islanded microgrids, Shafiee et al. [110] investigated distributed secondary control problem by a distributed networked control system which not only could be able to restore the frequency and voltage of the microgrid but also ensures reactive power sharing. It has also been shown that averaging-based distributed controllers using communication among the generation units offer the best combination of flexibility and performance in a microgrid [111].

### 5.2. Distributed Optimal Dispatch

With respect to power transmission systems, three kinds of control architectures, that is, a layering of primary, secondary, and tertiary control, have formed the standard operation paradigm for power systems. Generally, primary droop control is realized in a decentralized way, and secondary frequency regulation can be performed in a centralized, decentralized, or distributed architecture. Tertiary control is affiliated to unit commitment (UC) problem and economic dispatch (ED) problem, which are always solved in a centralized way. Owning to the explosion in size and complexity of modern electric power datasets, it is increasingly important to solve such an optimization problem high efficiently. Distributed convex optimization, in particular for large-scale problems arising in power systems, statistics, and other related areas, has received considerable attention recently.

Kar and Hug [112] proposed a distributed consensus-based approach for economic dispatch problem in power systems, in which each network agent participates in a collaborative process of neighborhood message exchange and local computation. By utilizing the distributed algorithm for frequency control and optimal economic dispatch of power generators, it was shown in [113] that distributed algorithm could eventually achieve optimality and present better robustness compared with traditional (centralized) dispatch algorithms. Economic dispatch problem was solved in distributed fashion by a novel consensus-based algorithm [114], where the estimated mismatch was used as a feedback mechanism to adjust current power generation by each generator such that all generators can automatically minimize the total cost in a collective sense. Yang et al. [115] considered the problem of distributed optimal dispatch based on the distributed primal-dual subgradient algorithm for virtual power plant (VPP). Based on two parallel consensus algorithms, a distributed algorithm was presented in [116] to solve the economic power dispatch problems with transmission line losses and generator constraints.

In fact, power systems can be modeled as a hybrid system, in which control and optimization coexist. Such as in the process of the frequency regulation, generating units and controllable flexible loads will participate in the secondary frequency regulation together. How the generators and loads respond to the frequency deviation is a cooperative control problem. While, for the tertiary frequency regulation problem, UC and ED problems are solved at the dispatching center traditionally; and distributed regulation and distributed optimization can be combined together to deal with such a multitime scale frequency regulation problem emerged in small or large power system.

## 6. Conclusion and Discussion

In this paper, we reviewed some recent progress in cooperative tracking control for leader-following multiagent systems and some relevant application problems in power systems by distributed regulation and optimization. Pinning control is an interesting research topic which has lots of potential applications. Although the existing theoretical results in distributed multiagent system are rich and varied, there are still many practical engineering problems to be solved which may involve the application of pinning control for large-scale interconnected systems. In the following, we mainly discussed some potential applications of pinning control in multiarea interconnected power system.

1. The interconnected power system can be modeled as a multiagent system, where network nodes are generators and buses, flexible loads, and network links are transmission/communication lines. While, in the power system, a basic tracking problem is the LFC problem [117, 118], in which the generators have to track the changing of active power of the time-varying loads, conventional control efforts focus on the generation side, mainly determined by AGC units, the objective of which is to control the reserved generation capacity and minimize the area control error (ACE) [119, 120]. In fact, such a control scheme is the centralized control; each generator receives the frequency regulation and generation scheduling signals from the dispatching center. Distributed pinning AGC may play an even greater advantage, which will be discussed in the near future.
2. Smart grids integrate different kinds of renewable power generations and multiple controllable flexible loads, which are connecting to the main power grid. These power generations together with AGC units and flexible loads will be involved in the primary frequency regulation of the power system together. So the basic problem comes of how do these participants coordinate with each other such that the frequency of power systems can be maintained at the normal operating range while subjected to the power balance constraints. The renewable power plants and AGC units can be categorized as the upper layer of generator cluster; flexible controllable loads are categorized as lower layer of consumer cluster. All units in each cluster can coordinate in a distributed way such that the frequency regulation is able to cope with the disturbance of the system.
3. The frequency regulation of power systems is accomplished through a three-stage process with the fast response primary control as the first stage. Secondary control can be contributed by all the generating units and controllable loads through distributed cooperative control, while controllable flexible loads in power systems are at a large scale of both size and distribution. For any given bus node, the loads under this bus can be aggregated to several clusters with each managed by a load agent. Is it necessary for loads to interact with each other inside a load agent and for agents to interact with each other under a bus node? It is urgent to introduce distributed or decentralized management instead of centralized management owing to the increasing number of managed objects. In the third-stage process, optimal dispatch can be performed at each generating/bus agent in a distributed manner. Thus, one can conclude that the frequency regulation of power systems is carried out by decentralized control in the first layer, distributed cooperative control in the second layer, and distributed optimization in the third layer, which makes the entire regulation system become more robust and efficient.
